# Size Constancy is Preserved but Afterimages are Prolonged in Typical Individuals with Higher Degrees of Self-Reported Autistic Traits

**DOI:** 10.1007/s10803-016-2971-6

**Published:** 2016-11-23

**Authors:** Irene Sperandio, Katy L. Unwin, Oriane Landry, Philippe A. Chouinard

**Affiliations:** 1 School of Psychology, University of East Anglia, Norwich Research Park, Norwich, NR4 7TJ UK; 2 School of Psychology, Cardiff University, Cardiff, Wales, UK; 3 Department of Psychology and Counselling, School of Psychology and Public Health, La Trobe University, Melbourne, VIC Australia

**Keywords:** Adaptation, Afterimage, Autism spectrum quotient, Light sensitivity

## Abstract

Deficits in perceptual constancies from early infancy have been proposed to contribute to autism and exacerbate its symptoms (Hellendoorn et al., Frontiers in Psychology 6:1–16, 2015). Here, we examined size constancy in adults from the general population (*N* = 106) with different levels of self-reported autistic traits using an approach based on negative afterimages. The afterimage strength, as indexed by duration and vividness, was also quantified. In opposition to the Hellendoorn and colleagues’ model, we were unable to demonstrate any kind of relationship between abilities in size constancy and autistic traits. However, our results demonstrated that individuals with higher degrees of autistic traits experienced more persistent afterimages. We discuss possible retinal and post-retinal explanations for prolonged afterimages in people with higher levels of autistic traits.

**Electronic supplementary material:**

The online version of this article (doi:10.1007/s10803-016-2971-6) contains supplementary material, which is available to authorized users.

## Introduction

Perceptual constancies are an integral aspect of our visual experience and critical to our successful interactions with the physical and social world. This is because they enable us to *see* objects as having the same physical properties despite changes in viewing conditions. If one were to view the world only by the image on the retina, it would appear distorted and unstable. For example, as we watch a car drive away from us, the size of its image on the retina would shrink as it speeds away. Although the car is shrinking on the retina, we perceive it exactly the same size, but just moving further away from us. This perceptual ability is known as size constancy. Size constancy is a scaling mechanism that allows us to make accurate judgments about an object’s size regardless of variations in the retinal image size arising from changes in viewing distance (Sperandio and Chouinard 2015).

Recently, Hellendoorn et al. (2015) proposed that problems from early infancy in size and other forms of perceptual constancy contribute to the development of autism. Specifically, the authors argued that infants who lack these skills see the physical world as less stable, which in turn creates greater levels of anxiety, exacerbating delays in understanding social and communication cues, and reinforces repetitive comfort-seeking behaviours towards familiar stimuli and tasks—all of which are features of autism. In line with this theory, Ropar and Mitchell (2002) demonstrated that shape constancy is affected in autism. Shape constancy is a perceptual mechanism that enables us to *see* 3D shapes as having the same geometry across different viewing conditions. Shape constancy is thought, much like size constancy, to rely on both bottom-up and top-down processes (for review, see Sperandio and Chouinard 2015), which are thought to be affected in autism according to the Enhanced Perceptual Functioning (EPF) (Mottron and Burack 2001; Mottron et al. 2006) and Weak Central Coherence (WCC) (Frith and Happé 1994; Happé and Frith 2006) theories.

We examined size constancy by measuring changes in perceived size of afterimages viewed at different distances (Emmert 1881; Sperandio et al. 2012; Sperandio and Chouinard 2015). An afterimage is an image attached to the retina that is experienced after a period of adaptation to a source of light. An afterimage appears like a shadow on a viewing surface; its perceived size increases proportionally as a function of the distance between the eyes and the viewing surface. This simple demonstration is formally known as Emmert’s law (Emmert 1881). It typically shows near-perfect size-scaling effects with changes in distance, such that when the distance between the eyes and the projected surface doubles the perceived size of the afterimage will double as well. Although it has been shown that size constancy with real objects and afterimages share similar neural underpinnings (Sperandio et al. 2012), working with afterimages offers the advantage to appreciate how processes taking place well beyond the retina affect size perception under conditions where there is absolutely no doubt that the retinal image remains constant.

In addition, the investigation of Emmert’s law gives us the opportunity to measure a number of other perceptual variables, such as duration and vividness of the afterimages. These experiences could be used to examine sensory abnormalities, such as hypersensitivity to light, which are now included in the more recent diagnostic criteria for autism [Diagnostic and Statistical Manual of Mental Disorders (DSM-5); American Psychiatric Association (APA) 2013]. It has been demonstrated that the duration of an afterimage generated by a light increases as a function of the amount of energy, intensity, and adaptation time of the light, corresponding presumably to the amount of bleaching in retinal pigment (Granit et al. 1930; Feinbloom 1938; Nagamata 1951; Alpen and Barr 1962). If the perceived duration of an afterimage is influenced by the intensity of the stimulus then one might expect those individuals with a higher sensitivity to light to experience prolonged afterimages. Hypersensitivity occurs when an individual’s perceived intensity to a stimulus is higher than it should be because of lower sensory thresholds. For example, a gentle touch can feel painful, noises may seem exceptionally loud, and lights may appear as unbearably bright in hypersensitive individuals (Dunn 1997). Hypersensitivity to light has been widely reported in autism (O’Leary et al. 1978; Attwood 1993; Schulman 1994; Williams 1994; Jones et al. 2003; Benezech and Chapenoire 2005; Bluestone and Brenner 2007; Coulter 2009; Simmons et al. 2009). In addition, a recent study has shown a strong relationship between the level of autistic traits and the frequency of atypical sensory behaviours in a typically developing population with 60% of the variance in sensory abnormalities explained by the variance in subclinical autistic traits (Robertson and Simmons 2013).

To our knowledge, no study has investigated the perception of afterimages induced by a bright source of light in autism before. The reasons for this gap in knowledge may relate to the risk of inducing discomfort in this population. Indeed, the presentation of a bright flash of light is reported to distress individuals with autism (e.g. Wing 1996). There is certainly anecdotal evidence from the autism community of persistent afterimages, among other visual disturbances (e.g. visual snow), following an exposure to bright sources of light. These visual disturbances in some cases can last for hours and be debilitating enough to interfere with daily activities, such as driving or walking outside on a sunny day (e.g. Jones et al. 2003).

Therefore, we opted to examine how abilities in size constancy and afterimage perception change as a function of autistic traits in a typically developing population. Levels of autistic traits were measured using three questionnaires: the Autism Spectrum Quotient (AQ, Baron-Cohen et al. 2001), the Systemizing Quotient (SQ, Baron-Cohen et al. 2003), and the Empathy Quotient (EQ, Baron-Cohen and Wheelwright 2004). The study of autistic traits within the typically developing population represents an alternative approach to a between-group design comparing individuals with and without autism.

Behavioural similarities between autism probands and unaffected family members have long been recognised (Kanner 1943) and a surge of more recent and genetic studies have documented the presence of autistic traits in relatives of individuals with autism (Bailey et al. 1995; Happe et al. 2001; Piven 2001; Sucksmith et al. 2011). These observations have lead to the development of a number of scales, including the three measures used in the present investigation, to quantify the degree to which any individual from the general population has autistic traits (for review, see Landry and Chouinard 2016). Behavioural investigations of unaffected family members and samples with higher scores on these scales often show similar patterns in perceptual and cognitive abilities as those seen in autism (e.g. Chouinard et al. 2013, 2016; Baron-Cohen and Hammer 1997; Bayliss and Tipper 2005; Bölte and Poustka 2006; Ruser et al. 2007; Whitehouse et al. 2007; Hudson et al. 2012; Palermo et al. 2006). Thus, relating autistic traits to performance on perceptual and cognitive tasks, not only provides insight into individual differences in the general population, but can also allow opportunities to examine phenomena that otherwise would be difficult to investigate properly in the autistic population due to confounding factors that are difficult to control for.

The present investigation had two aims. The first was to determine the integrity of size constancy abilities, by means of Emmert’s law, as a function of autistic traits in the typically developing population. The second was to determine if relationships exist between the strength of afterimages, as measured by their intensity and duration, and the degree of autistic traits in the typically developing population. We had two hypotheses—one for each of these aims. The first, on the basis of the model put forth by Hellendoorn et al. (2015), we predicted that the size of afterimages would deviate from Emmert’s law (Emmert 1881) in individuals with more autistic traits. Specifically, we predicted that the slope characterising the relationship between apparent size and viewing distance would deviate from a value of 1 as a function of autistic traits. The second, on the basis that many individuals with autism are hypersensitive to sensory stimulation, we predicted that vividness and duration of the afterimages would both increase as a function of autistic traits.

## Methods

### Participants

One hundred and six volunteers (65 males) ranging in age from 18 to 37 years of age (*M* = 21.27, *SD* = 3.68) took part in the experiment. All participants reported to have normal or corrected-to-normal visual acuity, and to have never been diagnosed with an autism spectrum disorder or any other neurological or psychiatric condition. Participants received either 4 study credits or £7 for their time. Written consent was obtained prior to testing. All procedures were approved by the local Institutional Review Board.

### Trait Questionnaires

To measure autistic traits, participants were asked to complete three questionnaires: the Autism Spectrum Quotient (AQ; Baron-Cohen et al. 2001), the Systemising Quotient (SQ; Baron-Cohen et al. 2003), and the Empathy Quotient (EQ; Baron-Cohen and Wheelwright 2004). The AQ is a self-report measure of autistic traits and is widely used in research (for a meta-analysis, see Ruzich et al. 2015). Its items can be grouped into five subscales: attention to detail, attention switching, imagination, communication, and social skill. Higher scores on the AQ indicate higher levels of autistic traits. The SQ and EQ are also self-report questionnaires (Baron-Cohen et al. 2003; Baron-Cohen and Wheelwright 2004). Higher scores on the SQ indicate more autistic traits whereas lower scores on the EQ indicate more autistic traits. All participants completed the AQ while 94 participants (53 males) completed the EQ and 94 (55 males) participants completed the SQ.

### Apparatus

**Fig. 1:**
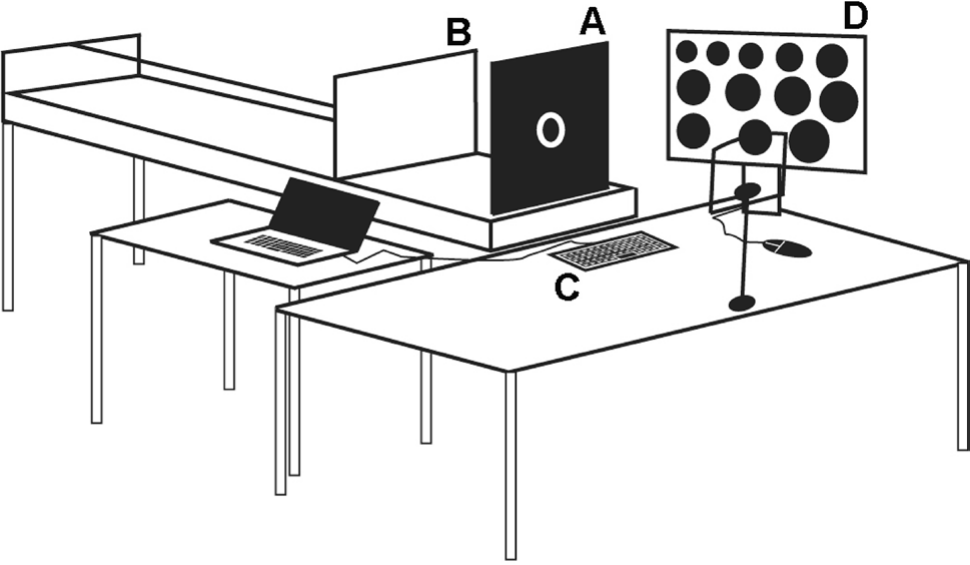
The experimental set up. *A* The collapsible front board with the LEDs. *B* The movable back board. *C* The keyboard used to record the participant’s response. *D* The reference circles for the size-matching task

The experiment was programmed in E-Prime 2.0 Professional software (Psychology Software Tools, Pittsburgh, PA) on an Acer Travel Mate 240 laptop running Windows XP. Negative afterimages were induced by means of a ring of white light emitting diodes (LEDs) with a luminance of 2686 cd/m^2^ as described in Sperandio et al. (2013). The inducing stimulus light was centrally mounted into a collapsible wooden panel painted with black matte paint. The light was positioned 69.8 cm away from the participant’s eyes and subtended 4.1° of visual angle. An apparatus similar to Sperandio, Chouinard and Goodale (2012) was used to manipulate viewing distance. The apparatus consisted of a front board that had a set of LEDs mounted to it and a white back board that could be slid to different viewing distances using a wooden dowel. Five viewing distances were tested: 107.2, 139.7, 172.1, 204.6, and 237 cm. To obtain judgments of perceived size, a Dell TFT 23 inch UltraSharp computer monitor (1920 × 1080 pixels) positioned at a viewing distance of 57 cm was used to display 38 reference circles. Participants matched the size of the afterimage they saw at a specific viewing distance with one of these circles. The circles’ diameter ranged from 5.2 cm (5.2° of visual angle) to 19.4 cm (19.4° of visual angle) in increments of 0.4 cm (0.4° of visual angle) (Fig. 1). The circles’ diameter was selected to exceed the range of theoretical sizes of afterimages as specified by Emmert’s law (Sperandio et al. 2013).

### Procedure

The procedures for inducing and measuring afterimages are almost identical to those used previously by Sperandio et al. (2012, 2013). The participant sat in front of a table in a dimly lit room with their chin on a chin-rest. The height of the chin-rest was adjusted to align their line of sight with the centre of the LEDs. The experiment included 25 trials (5 trials × 5 viewing distances). The order of trials was randomly generated by E-Prime. At the beginning of each trial, the experimenter placed the front board housing the inducing light in the upright position and the participant looked directly at the light with a steady gaze. Meanwhile, the experimenter received instructions via headphones regarding one of the five possible viewing distances and placed the back board at the cued position. The light was then turned on for 200 ms to induce an afterimage. As soon as the light was turned off, an acoustic warning (i.e. a ‘beep’) was delivered to the experimenter through headphones to promptly fold the front board flat and reveal the back board. This allowed the participant to ‘project’ their afterimage onto the back board. Once the afterimage disappeared completely, the participant pressed the space bar on the keyboard. Next, they performed the size-matching task, by choosing one of the reference circles presented on a monitor (Fig. 1D) that corresponded to the remembered size of the afterimage seen at a particular viewing distance. Finally, the participant judged the vividness of their afterimage on a scale from zero to nine with zero meaning “no afterimage whatsoever” and nine meaning “a very vivid and clear afterimage, like a real stimulus”. Judgments of vividness were based on the remembered clarity of the afterimage seen at a specific viewing distance. To enable the participant to familiarise themselves with the procedures, a minimum of three practice trials were provided before testing began.

### Data Analysis

We carried out all statistical analyses using SPSS (IBM Corporation; Armonk, New York, USA). Unless specified otherwise, all reported *p* values were based on two-tailed criteria and corrected for multiple comparisons using the Bonferroni method (i.e. *p*_*corr*_ = *p*_*uncorr*_ × total number of comparisons; Dunn 1961). To examine the integrity of size constancy mechanisms, a measurement of theoretical size according to Emmert’s law was first calculated using the following equation:

$$s=d\times \tan (\theta )$$

whereby the theoretical size (s) of the perceived afterimage was equal to the distance (d) the afterimage was viewed multiplied by the visual angle (tan, θ) subtended by the afterimage. After this was performed, a correlation between subjective ratings of perceived and theoretical size was carried out and a linear regression analysis was performed to compute the slope.

Mean duration and mean vividness of the afterimages were also calculated. Pearson’s correlation coefficients (*r*) were calculated between each measure of the afterimage (i.e. size slope, duration, and vividness) and each of the quotient scores (i.e. AQ, EQ, and SQ), as well as between each of the AQ subscales. Finally, a median-split approach was used to compare each perceptual report of the afterimage (i.e. size slope, duration and vividness) between participants with low versus high scores on the AQ, EQ, and SQ questionnaires. Results from this additional analysis are reported in Supplementary Materials.

## Results

**Fig. 2:**
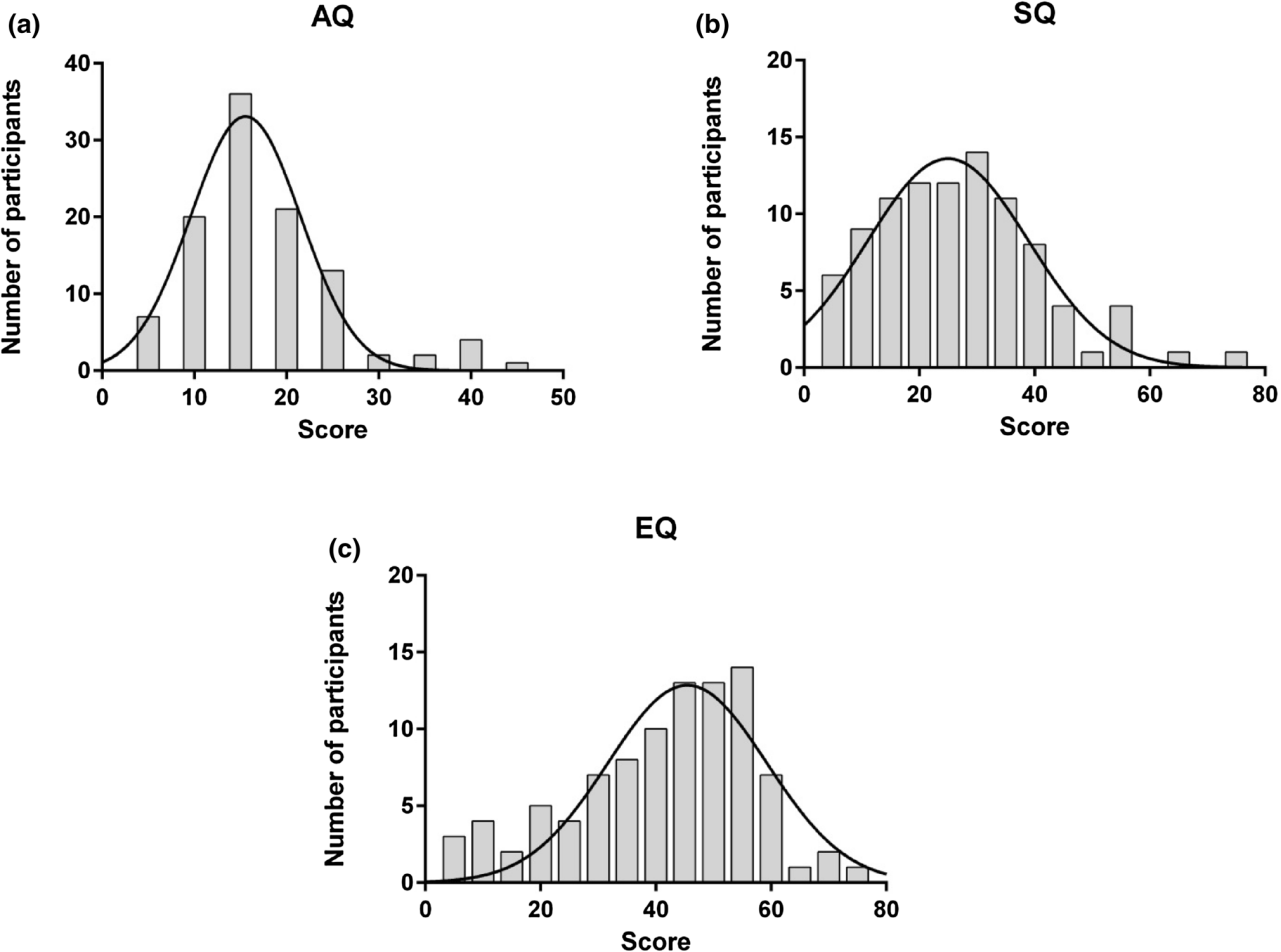
Distributions for AQ (**a**), SQ (**b**), and EQ (**c**) scores. The* x-axis* corresponds to the scores while the* y-axis* corresponds to number of participants. The *solid lines* denote Gaussian functions that best fit each distribution

The distributions for AQ, EQ, and SQ are reported in Fig. 2. The AQ scores were distributed with a mean of 19.85, a standard deviation of 8.02, and a range of 4–43. Such a distribution is typical for a non-clinical population (e.g. Ruzich et al. 2015). The SQ scores were distributed with a mean of 38.45, a standard deviation of 14.12, and a range of 5–74. The EQ scores were distributed with a mean of 38.85, a standard deviation of 15.43, and a range of 5–73. Such distributions for both SQ and EQ are also typical for a non-clinical population (e.g. Groen et al. 2015). Pearson’s correlation revealed significant relationships between each pair of questionnaires (all *p* < .001) in the expected direction: a positive correlation between AQ and SQ (*r*_*(92)*_ = .55) and negative correlations between AQ and EQ (*r*_*(92)*_ = −.64) as well as SQ and EQ (*r*_*(92)*_ = −.39).

### Relationship Between Autistic Traits and Size Constancy

**Fig. 3:**
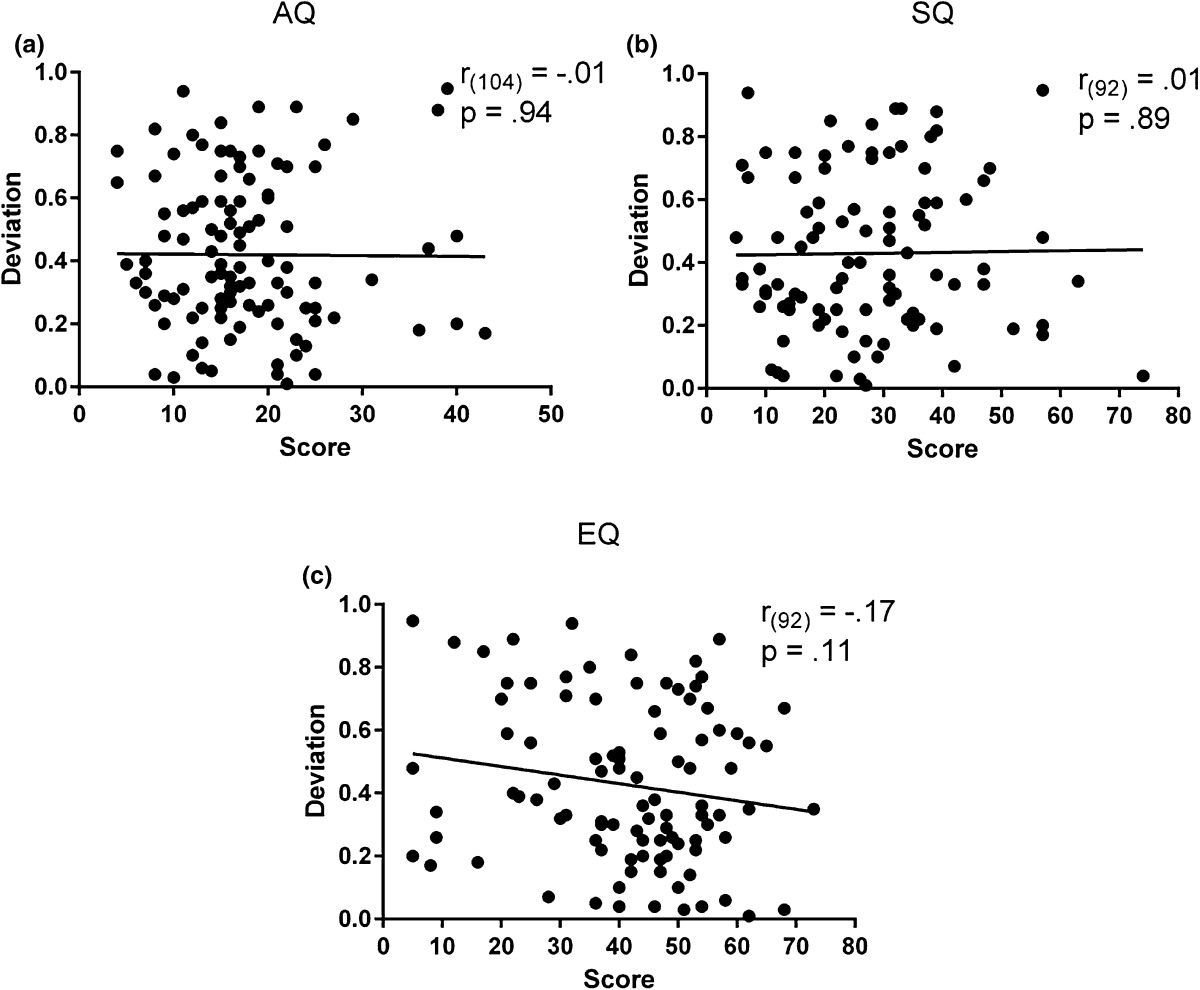
Correlations between deviation values and the AQ (**a**), SQ (**b**), and EQ (**c**) scores. The lack of association between deviations and questionnaires’ scores indicate that size constancy mechanisms are unaffected by autistic traits. The *x-axis* corresponds to the scores while the *y-axis* corresponds to the absolute deviations from Emmert’s law. Pearson correlation coefficients (*r*) and the corresponding *p* values (uncorrected) are reported in each panel

To evaluate the extent to which the changes in afterimage size followed Emmert’s law (Emmert 1881), we first correlated the subjective ratings of the size of the afterimages with their predicted size for each viewing distance. Overall, participants’ estimates of perceived size closely followed Emmert’s law (*M* = 0.96, *SD* = 0.07). Only 5 participants out of 106 (4.7%) did not show a significant relationship with Emmert’s law (see Figure S1 in Supplementary Materials for examples of individual data). Then, we calculated the slope of the regression line. A slope of 1 would indicate perfect size-distance scaling whereas a shallower or steeper slope would indicate less adherence to Emmert’s law. The slope values (*M* = 0.58, *SD* = 0.25) were then correlated with quotient scores (see Figure S2 in Supplementary Materials). The correlation revealed that there was no significant relationship between slope size and AQ (*r*_*(104)*_ = −.001, *p*_*uncorr*_ = .99), SQ (*r*_*(92)*_ = −.02, *p*_*uncorr*_ = .81), or EQ (*r*_*(92)*_ = .14, *p*_*uncorr*_ = .17). In addition, the amount of deviation of the slope from perfect size constancy was computed for each participant as an absolute difference between the slope value and 1. The deviation values (*M* = 0.42, *SD* = 0.24) were then correlated with quotient scores. Once again, the correlation revealed that there was no significant relationship between slope size and AQ (*r*_*(104)*_ = −.01, *p*_*uncorr*_ = .94), SQ (*r*_*(92)*_ = .01, *p*_*uncorr*_ = .89), or EQ (*r*_*(92)*_ = −.17, *p*_*uncorr*_ = .11). Therefore, the results show that size constancy is unaffected by autistic traits (Fig. 3).

### Relationship Between Autistic Traits and Afterimage Duration

**Fig. 4:**
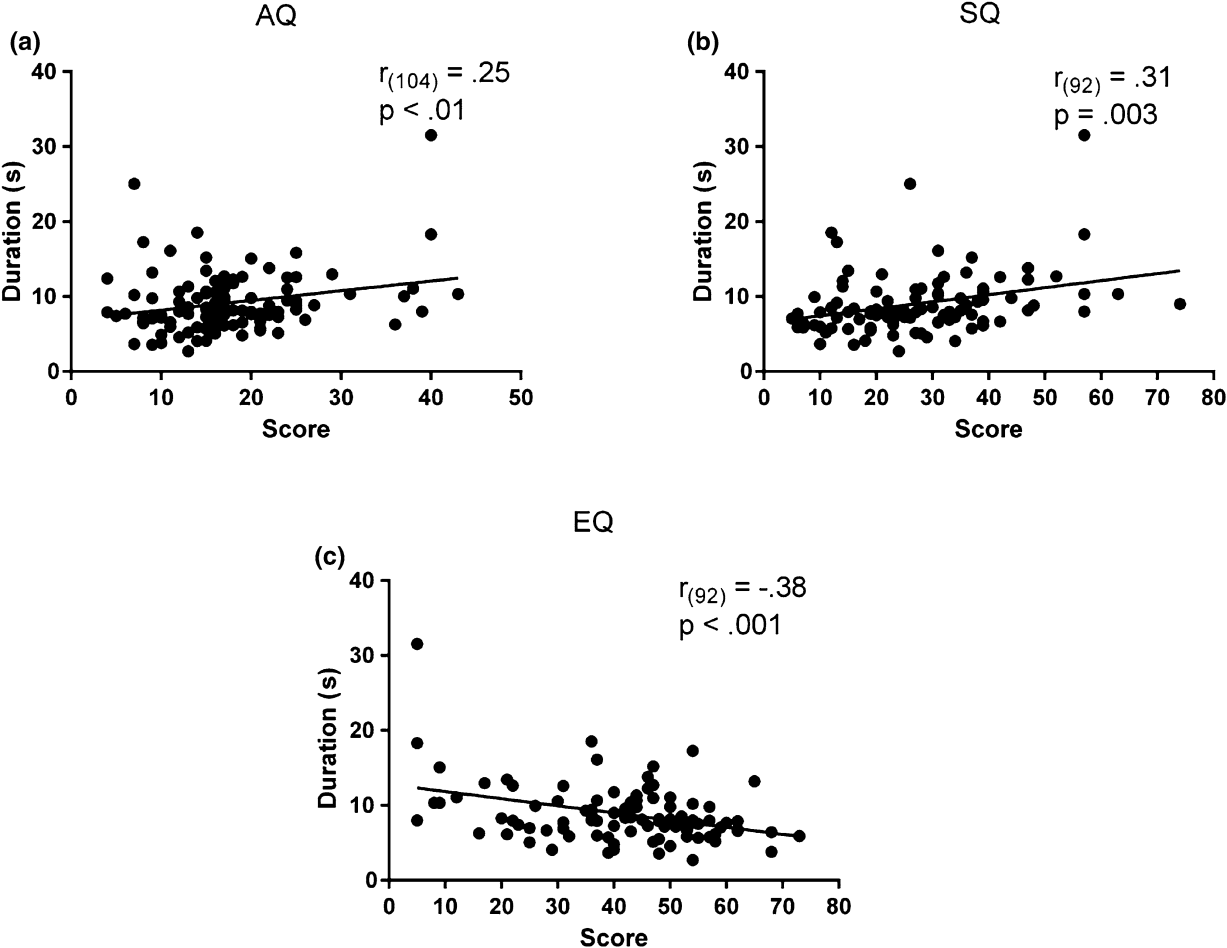
Correlations between the perceived duration of the afterimage and the AQ (**a**), SQ (**b**), and EQ (**c**) scores. The positive correlations for AQ and SQ (**a, b**) and the negative correlation for EQ (**c**) show how individuals with elevated autistic tendencies experienced prolonged afterimages. The* x-axis* corresponds to the scores while the* y-axis* corresponds to the duration of the afterimages in ms. Pearson correlation coefficients (*r*) and the corresponding *p* values (uncorrected) are reported in each panel

The mean duration of the afterimage was correlated with AQ, SQ and EQ scores. Namely, the perceived duration of the afterimage correlated positively with AQ (*r*_*(104)*_ = .25, *p*_*uncorr*_ = .01) and SQ (*r*_*(92)*_ = .31, *p*_*uncorr*_ = .003) scores, and negatively with EQ (*r*_*(92)*_ = −.38, *p*_uncorr_ < .001) scores. That is, the higher the degree of autistic traits, the longer the duration of the afterimage (Fig. 4).

To further investigate the relationship between afterimage duration and autistic traits, the perceived afterimage duration was correlated with scores from the different subcategories of the AQ. One-tailed criteria were applied given we had already demonstrated a positive correlation with total AQ. Attention switching and communication were positively correlated with perceived afterimage duration (*r*_*(104)*_ = .26, *p*_*corr*_ = .02; *r*_*(104)*_ = .27, *p*_*corr*_ = .01, respectively) whereas social skill (*r*_*(104)*_ = .16, *p*_*corr*_ = .3), attention to detail (*r*_*(104)*_ = .13, *p*_*corr*_ = .46), and imagination (*r*_*(104)*_ = .1, *p*_*corr*_ = .76) were not correlated with perceived afterimage duration. These findings indicate that those participants with higher autistic traits related to attention switching and communication experienced longer afterimages.

### Relationship Between Autistic Traits and Afterimage Vividness

**Fig. 5:**
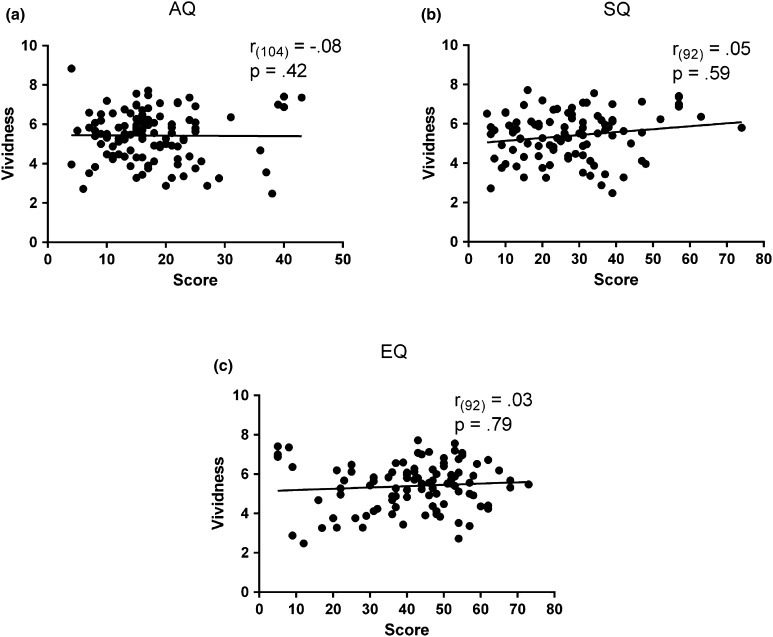
Correlations between vividness and the AQ (**a**), SQ (**b**), and EQ (**c**) scores. The lack of correlations between vividness and questionnaires’ scores indicate that afterimage visibility is unaffected by autistic traits. The* x-axis* corresponds to the scores while the* y-axis* corresponds to the vividness scores (range 0–9). Pearson correlation coefficients (*r*) and the corresponding *p* values (uncorrected) are reported in each panel

All participants reported an afterimage on every trial. On average, afterimages were reliably perceived by the participants (*M* = 5.43, *SD* = 2.77). Vividness did not correlate with AQ (*r*_*(104)*_ = −.08, *p*_*uncorr*_ = .42), SQ (*r*_*(92)*_ = .05, *p*_*uncorr*_ = .59), or EQ (*r*_*(92)*_ = .03, *p*_*uncorr*_ = .79) score, suggesting that the level of autistic traits did not affect the visibility of the afterimage (Fig. 5).

### Relationship Between Afterimage Duration and Vividness

To verify if afterimage visibility affected perceived duration, vividness scores were correlated with duration. As it turned out, vividness did not correlate with duration (*r*_*(104)*_ = .11, *p* = .25). We argue that the lack of relationship between these two perceptual attributes of the afterimage may be due to a reduced sensitivity in measuring vividness. In fact, vividness ratings were exclusively based on memory processes. This was not the case for afterimage duration. As a consequence, ratings of vividness were more subjective than judgments about duration.

## Discussion

We characterised the effects of autistic traits in the general population on size constancy and afterimage strength. The investigation of size constancy is timely in light of a recent account by Hellendoorn et al. (2015). This theory attempts to explain a variety of autistic behaviours in terms of deficits in either the formation or application of invariant representations. These representations are critical for perceiving an external world as stable and predictable regardless of changes in retinal input and promoting feelings of security. Based on the theory, one might expect that size constancy mechanisms would also be altered in those with higher autistic traits. In the ensuing discussion, we first provide a summary of the present findings on the relationships between autistic traits and size constancy as well as between autistic traits and afterimage strength (i.e. duration and vividness). Next, we discuss the possible link between hypersensitivity and autistic traits in the general population. Finally, we consider what factors might affect the perceived duration of an afterimage and suggest possible avenues for future research.

### The Integrity of Emmert’s Law did not Vary as a Function of Autistic Traits

We assessed size constancy by means of afterimages viewed at different distances. Under these conditions, the perceived size of an afterimage changes linearly with distance, as predicted by Emmert’s law (Emmert 1881). Contrary to what one might predict from Hellendoorn et al. (2015) theory, we did not observe any relationships between AQ, EQ or SQ score and size-distance scaling, suggesting that size constancy mechanisms are unaffected by autistic traits. Therefore, our results demonstrate that size constancy, a basic perceptual mechanism that allows invariant representations of size, operates normally in those with higher degrees of autistic traits. To our knowledge, the presence of this association has never been tested before.

### Persistence of Afterimages Increased as a Function of Autistic Traits

We quantified afterimage strength in terms of its duration and vividness. We predicted that those with higher autistic traits would experience stronger afterimages, based on the evidence that many individuals on the autism spectrum are hypersensitive to light (O’Leary et al. 1978; Attwood 1993; Schulman 1994; Williams 1994; Jones et al. 2003; Benezech and Chapenoire 2005; Bluestone and Brenner 2007; Coulter 2009; Simmons et al. 2009). Our results supported this prediction. The duration of the afterimage correlated positively with AQ and SQ, and correlated negatively with EQ. However, the results also revealed that autistic traits did not influence the reported vividness of the afterimages. Such a finding is in agreement with a recent study on colour adaptation to photographs of scenes by Maule et al. (2016), where no difference in colour afterimage strength between those with autism and typical individuals was reported.

### AQ Subscales and Afterimage Duration

The assessment of which specific subscales within the AQ correlate with afterimage duration can provide insight into which cognitive aspects associated with autism may be directly related to the effects observed with overall AQ scores. The Attention Switching and Communication subscales of the AQ accounted for the prolonged duration of the afterimages.

To account for the former, we postulate that there might be a link between prolonged duration afterimages and difficulties in attention switching. Higher scores on the Attention Switching subscale mean greater perseveration, which is frequently reported in autism (Landry and Al-Taie 2016). As it turns out, the more a person attends to an afterimage after it appears, the faster it will disappear (Lou 2001). In the present investigation, we instructed participants to first attend to the inducing light and then project their afterimage to a back board. It is conceivable that attention would switch from this fixation light to an afterimage after it appears and that this switch in attention would be slower in people with higher scores on the Attention Switching subscale, prolonging the duration of the afterimages.

Indeed, abilities to shift attention are affected in autism. Landry and Parker (2013) systematically examined the literature on spatial shifts of attention in autism. Combining a total of 18 papers, their meta-analysis revealed that individuals with autism demonstrated a moderate impairment in spatial shifts of attention when compared to individuals without autism (*Cohen’s d* = 0.44). It could be the case that hypersentivity to sensory stimuli may hinder a person’s volition to shift towards these same stimuli. It is also conceivable that this hindrance may have undesirable consequences during development. Spatial shifting of orientation emerges in early infancy and is thought to be important for the development of communication (Johnson and De Haan 2015). Delays in shifting attention, such as in autism, could therefore lead to delays in understanding language cues, exacerbating symptoms and problems in abilities to communicate, and contributing to life-long negative outcomes. This may explain why the Communication subscale of the AQ also correlated with afterimage duration.

### Relationship Between Hypersensitivity and Autistic Traits in the General Population

Our results reveal how hypersensitivity to light, as indexed by the duration of afterimages, increases as a function of autistic traits. Sensory sensitivities, such as hypersensitivity to sensory input, have been found in autistic populations (for review see, Baum et al. 2015) and now form part of the diagnostic criteria for autism spectrum disorder (APA 2013). These sensory sensitivities have also been found in a sub-clinical autism population. For instance, Horder et al. (2014) have shown that autistic traits (in both a clinical and a sub-clinical population, *n* = 772) correlated positively with scores on the Glasgow Sensory Questionnaire (GSQ; Robertson and Simmons 2013), which measures abnormal sensory symptoms. Robertson and Simmons (2013) also found that those with higher autistic traits (as measured by the AQ) reported higher levels of sensory hypersensitivity (as measured by the GSQ), suggesting that individuals in the general population with higher levels of autistic traits are characterized by sensory hypersensitivities.

### Retinal Explanations for Prolonged Afterimages

The photoreceptor cells in the retina (i.e. the rods and cones) require photochemical reactions of rhodopsin to properly transduce light energy into a neural signal. These photochemical changes occur every time the photoreceptor cells capture and convert light into a neural signal. When light stimulation reaches a certain point, the rhodopsin in the photoreceptor cells gets depleted and photoreceptors are no longer responsive to light until the rhodopsin has been restored. It is known that this photochemical bleaching plays a necessary role in the creation of afterimages (e.g. Feinbloom 1938; Brindley 1962; Williams and Macleod 1979). Hence, possible explanations for the prolonged afterimages in individuals with higher levels of autistic traits could relate to various factors mediating the degree to which photoreceptor cells could get bleached such as (1) how levels of light entering the pupil is regulated, and (2) how the oculomotor system regulates eye movements such that the same parts of the retina do not become over-stimulated.

Regarding the first possibility, atypical pupillary light reflex (PLR) has been observed in children with autism (Rubin 1961; Fan et al. 2009; Daluwatte et al. 2013) and their siblings (Nyström et al. 2015), which some have argued could be used as a biomarker of autism. The PLR regulates the light flux that enters the eye to the retina, playing a role similar to the aperture of a camera. When a flash of light is detected by the retina, the pupil will undergo an initial constriction and then recover once the flash is removed. Pupillometry studies in children with autism have revealed slower pupillary constriction (Rubin 1961; Fan et al. 2009), longer PLR latency, reduced constriction amplitude (Fan et al. 2009; Daluwatte et al. 2013), and shorter constriction/re-dilation time (Daluwatte et al. 2013) in response to light compared to neurotypical controls. Abnormal PLR has been reported as a predictor of atypical sensory behaviours in children with autism (Daluwatte et al. 2015). Importantly, the perception of an afterimage is accompanied by pupillary activity that is modulated by the intensity and duration of the primary stimulus (Newsome 1971; Alpern and Ohba 1972). Future investigations should examine if abnormal PLR can explain the persistent afterimages observed in those with high autistic traits.

Regarding the second possibility, previous research has shown that saccadic eye movements reduce retinotopic adaptation, generating shorter afterimages (Bachy and Zaidi 2014). Based on this assumption, along with the present findings, it can be hypothesised that those with higher autistic-like traits might fixate better than those with lower autistic-like traits during the induction of the afterimage and thereby perceive a longer afterimage. Whilst there is evidence of elevated saccadic frequency in children with autism during and between visual tasks that do not require fixation (e.g. Kemner et al. 1998), participants in the present study were asked to fixate their gaze at one location (i.e. the light). Interestingly, a recent study on fixation stability in adults with autism has shown difficulty in maintaining steady gaze especially in the absence of a fixation target, providing evidence for abnormalities in ocular fixation control but only when there was no target (Shirama et al. 2016). It is also known that some people with autism sometimes stare at objects that interest them, blocking all else out of their attention. In the majority of these cases, the presence of staring episodes is not indicative of epilepsy or other comorbid neurological disorder (Hughes et al. 2015). Further investigation, measuring micro-saccades and pupil size during conditions of prolonged fixation, would be required to determine if an association exists between fixation stability and autistic traits and how this can affect the perceived duration of afterimages.

### Post-retinal Explanations for Prolonged Afterimages

Although retinal adaptation is required to induce an afterimage, post-retinal factors also play an important role in afterimages. Indeed, it has been reported that afterimages can be affected by higher cognitive operations, such as perceptual filling (Shimojo et al. 2001; van Lier et al. 2009), attention (Suzuki and Grabowecky 2003; van Boxtel et al. 2010), awareness (van Boxtel et al. 2010), and contextual integration (Sperandio et al. 2012)—suggesting cortical contributions to the perceptual phenomenon of afterimages. In an fMRI study, Sperandio, Chouinard and Goodale (2012) confirmed the importance of cortical processing in the generation of afterimages by demonstrating increases in both the magnitude and duration of the BOLD response in the primary visual cortex as a function of afterimage duration. This leads us to speculate that those with higher autistic traits might exhibit cortical hyper-excitability to light stimulation. However, further investigation is required.

Post-retinal processing is thought to be critical in explaining why afterimages are rarely experienced in everyday life, despite the fact that we are constantly exposed to stimuli that are bright enough to induce adaptation, such as staring at a computer screen or being exposed to bright lights while driving a car at night. According to one account, afterimages are ambiguous signals for the brain to process, which could be interpreted as being either a real object or a retinal artefact (i.e. an afterimage) (Powell et al. 2012). A contextual cue that the brain might use to resolve this problem is whether or not the stimulus remains fixed on the retina, given that real objects do not move with the eyes and if an object remains fixed on the retina then it must be an artefact of the eye (Powell et al. 2015). This account may explain why saccadic eye movements decrease afterimage duration as they may provide evidence against the afterimage being a real object (e.g. Fiorentini and Mazzantini 1965; Powell et al. 2015). A number of studies demonstrate that typically developing individuals tend to show a perceptual style privileging local details over global integration as function of autistic traits (e.g. Sutherland and Crewther 2010). The present investigation shows how individuals with more autistic traits also tend to demonstrate persistence in afterimages. It could be the case that afterimages are prolonged as a function of autistic traits because of co-varying differences in the processing of contextual elements that should signal the brain to interpret the retinal noise as an afterimage as opposed to a real object.

Over the last few years a Bayesian account of autism to explain atypical sensory processing has started to gain considerable attention (e.g. Pellicano and Burr 2012; Lawson et al. 2014; Sinha et al. 2014; Rosenberg et al. 2015). Specifically, this theory proposes that what is disrupted in autism is not the sensory processing itself, but the interpretation of the sensory input, whereby internal priors are under-weighted and less used. Following this logic, one could also explain the prolonged afterimages in individuals with higher autistic traits, as shown here, to occur as a result of weaker priors alerting the brain that the afterimage is not a real object and thus should be suppressed. Further investigation is necessary to better understand the neural mechanisms responsible for afterimage perception and their relationship with hypersensitivity to light.

### Closing Remarks

Perceiving the world in a stable and predictable manner is fundamental to day-to-day interactions with our surroundings and other people. Nevertheless, perceptual constancies in autism are still a neglected topic in the literature. Here, we show for the first time that size constancy mechanisms, as assessed by afterimages, operate normally in individuals with higher levels of autistic traits. Another important conclusion to draw from the current study is that afterimage duration increases as a function of the continuum of autistic traits in the general population. The prolonged afterimages experienced by those with high levels of autistic traits might be linked to some of the sensory difficulties experienced by those with autism, specifically hypersensitivity to light. An important limitation of the current investigation is that we did not include a measure of sensory sensitivity or abnormal sensory experiences, such as the Sensory Perception Quotient (SPQ, Tavassoli et al. 2014), the Adult/Adolescent Sensory Profile (AASP, Brown and Dunn 2002) and the Cardiff Anomalous Perceptions Scale (CAPS, Bell et al. 2006), which would allow a more direct assessment of the relationship between afterimage perception and sensory hypersensitivity. Another fundamental limitation is that we did not test individuals with autism directly. Therefore, it is possible that repeating the same procedures on this population might lead to different conclusions. However, our approach offers the clear advantage of controlling for confounding variables, including co-morbid disorders, symptom severity, cognitive ability, and variable compliance, which make it difficult to carry out well-controlled visual psychophysical experiments in samples with autism, while reducing the risk of inducing unpleasant experiences from presenting bright lights in a population that is known to be hypersensitive to sensory stimulation. Finally, one may argue we had poor control over how we carried out the procedures for measuring afterimage duration in terms of timing. Although there is room for improvement (e.g. having everything automated robotically would ensure millisecond accuracy in terms of timing presentation and would conceivably reduce signal to noise ratio in the data), we simply did not have the resources to do this. Nevertheless, Sperandio, Chouinard and Goodale (2012) showed how almost identical procedures for measuring afterimage duration can explain >80% variability in the fMRI activation in the primary visual cortex—demonstrating how the procedures we used here were certainly sensitive and powerful enough for detecting an effect should an effect exist. Furthermore, the present investigation demonstrated that afterimage duration did in fact correlate with AQ, EQ, and SQ. We would not have obtained these results had our procedures not been sensitive enough.

## Electronic supplementary material

Below is the link to the electronic supplementary material.

Supplementary material 1 (DOCX 243 KB)
