# Supplementary material for: Size Constancy is Preserved but Afterimages are Prolonged in Typical Individuals with Higher Degrees of Self-Reported Autistic Traits
Source: J Autism Dev Disord. 2016 Nov 23;47(2):447–59. doi: 10.1007/s10803-016-2971-6 (PMC5309324; doi:10.1007/s10803-016-2971-6)
Supplement: Supplementary file 1 — Supplementary material 1 (DOCX 243 KB) [file 10803_2016_2971_MOESM1_ESM.docx]

*Relationship between perceived and predicted size of the afterimage*

*
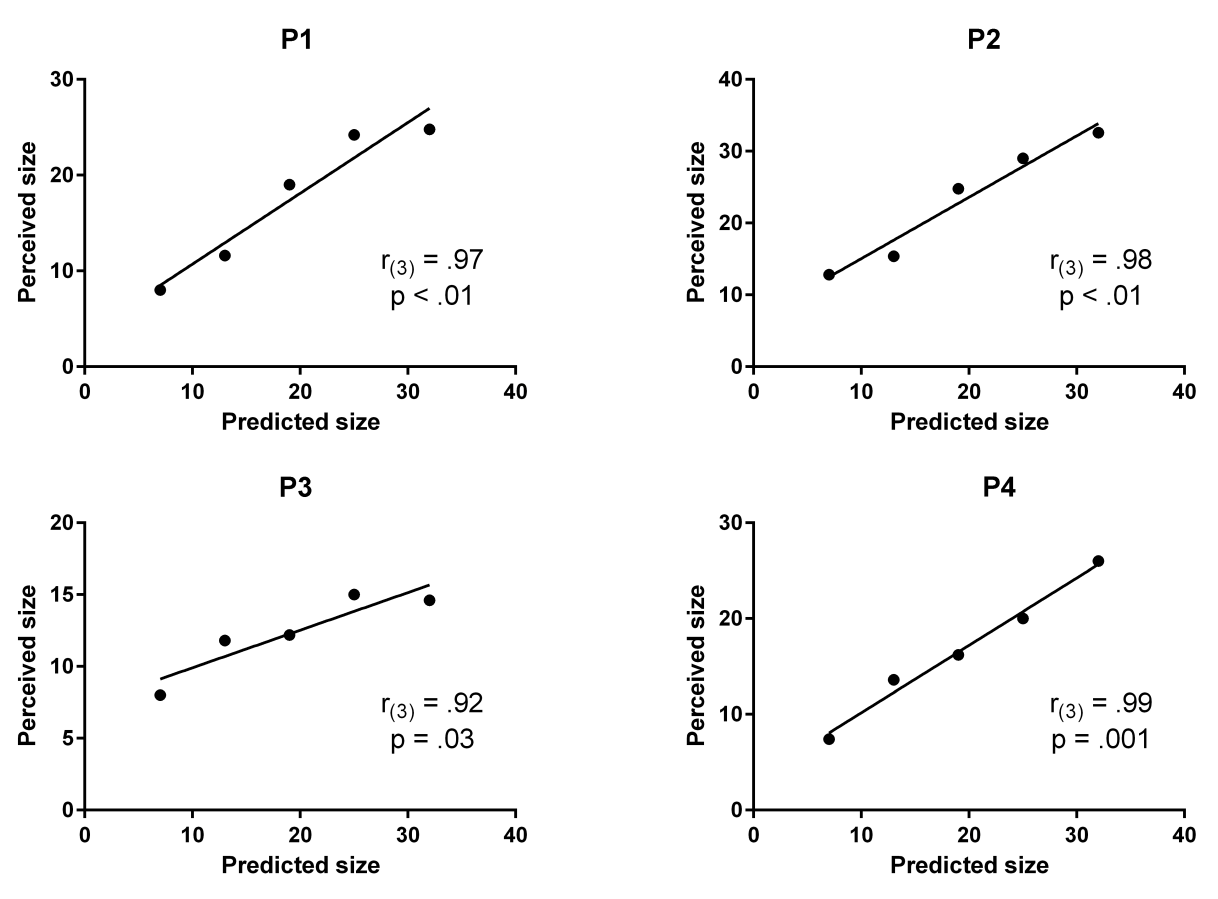
*

*Figure S1.* Examples of individual data from four participants (P1; P2; P3; P4). Correlations between perceived size and theoretical size based on Emmert's law. The x-axis corresponds to the predicted size while the y-axis corresponds to the observed size. Pearson correlation coefficients (*r*) and the corresponding *p* values are reported in each panel.

*Relationship between autistic traits and size constancy*

*
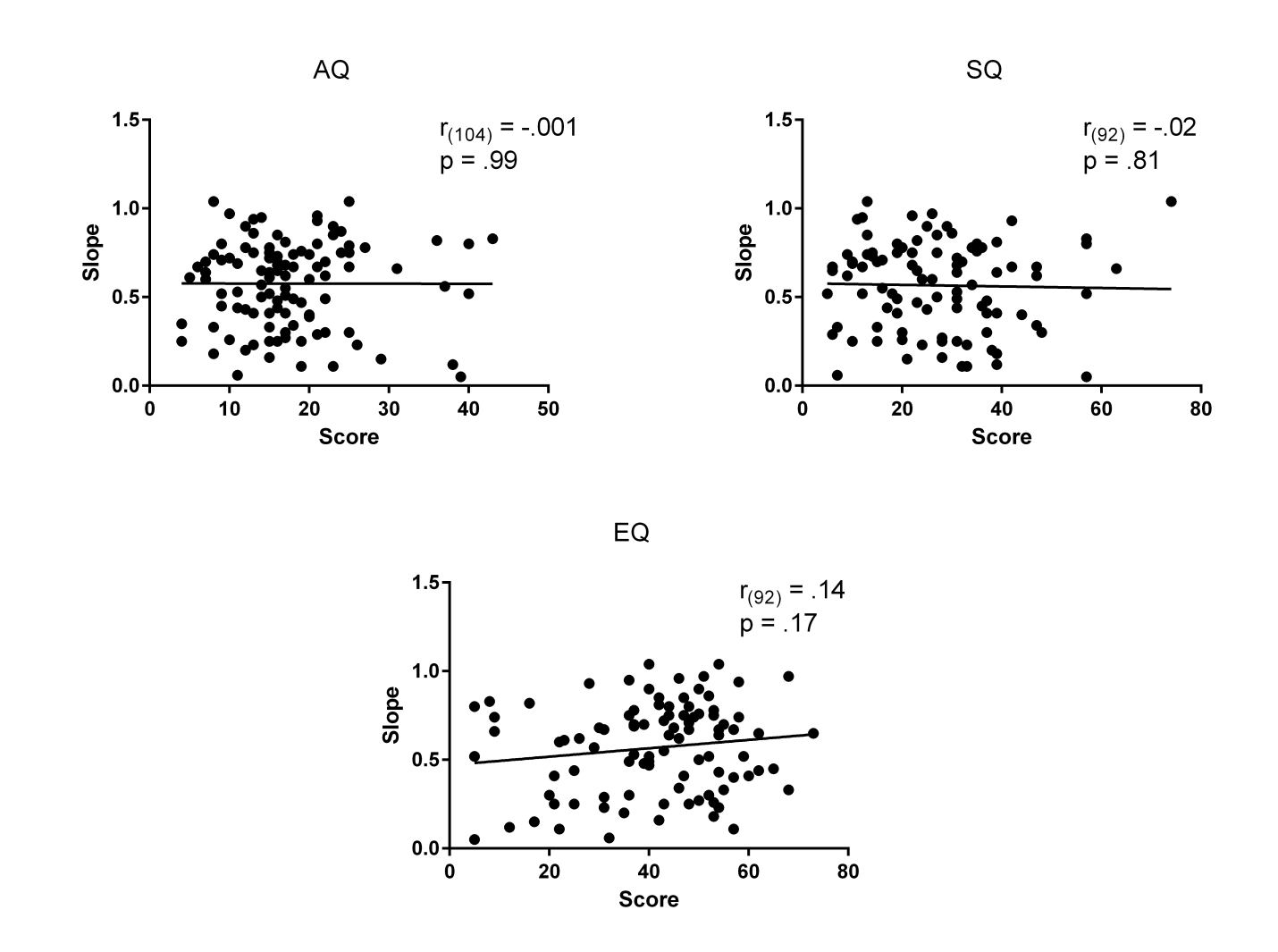
*

*Figure S2.* Correlations between slope values and the AQ (a), SQ (b), and EQ (c) scores. The lack of association between slopes and questionnaires' scores indicate that size constancy mechanisms are unaffected by autistic traits. The x-axis corresponds to the scores while the y-axis corresponds to the observed slopes. Pearson correlation coefficients (*r*) and the corresponding *p* values (uncorrected) are reported in each panel.

*Results based on a median-split approach*

In disagreement with the regression-based approach, the median-split approach did not generate any significant findings. The median score for AQ was 16, the median score for SQ was 26.5 and the median score for EQ was 43.5. Independent samples t-tests did not reveal any significant differences between the low (*n* = 51) and high (*n* = 55) AQ groups for size slope (*t*_(104)_ = -.07, *p* = .94), duration (*t*_(104)_ = -1.44, *p* = .15), and vividness (*t*_(104)_ = .82, *p* = .41). Additional independent samples t-tests did not show any significant differences between the low (*n* = 61) and high (*n* = 60) SQ groups for size slope (*t*_(92)_ = 1.34, *p* = .18), duration (*t*_(92)_ = -1.90, *p* = .06), and vividness (*t*_(92)_ = -.79, *p* = .43). Likewise, no significant differences were observed between the low (*n* = 47) and high (*n* = 47) EQ groups for size slope (*t*_(92)_ = -1.34, *p* = .18), duration (*t*_(92)_ = 1.80, *p* = .07), and vividness (*t*_(92)_ = -.75, *p* = .46). We attribute the lack of effects to the reduced sensitivity of the median-split approach compared with the regression-based approach (for further discussion, see MacCallum, Zhang, Preacher, & Rucker 2002).

**References**

MacCallum, R. C., Zhang, S., Preacher, K. J., & Rucker, D. D. (2002). On the practice of dichotomization of quantitative variables. *Psychological Methods*, 7(1), 19-40.
